# Hypotheses on physiopathogenic mechanisms of fecal incontinence in Wolfram syndrome

**DOI:** 10.1016/j.ibneur.2026.07.024

**Published:** 2026-08-12

**Authors:** C. Orssaud

**Affiliations:** Functional Unit of Ophthalmology, ERN Eye, Ophthalmological Rare Diseases Center, Georges Pompidou European Hospital, 20 rue Leblanc, Paris 75015, France

**Keywords:** Autonomic disorders, Fecal incontinence, Urinary disorders, Wolfram syndrome

## Abstract

Type 1 Wolfram syndrome is a rare, neurodegenerative pathology characterized by the association of insulin-dependent diabetes and optic atrophy. Other symptoms include diabetes insipidus, hearing loss and neurological damage. Fecal incontinence was recently recognized as a new symptom. Its pathophysiological mechanism remains unknown. We plan to collect data on the severity of the syndrome at the onset of fecal incontinence, the frequency of signs suggestive of pons volume reduction, and the frequency of urinary disturbances. Based on this information, we will investigate the possible location of the neurological involvement causing this symptom.

## Highlight

• Fecal incontinence was recently recognized as a new symptom of type I Wolfram syndrome.Four hypotheses will be tested in order to determine its pathophysiological mechanism.

## Introduction

Wolfram syndrome (WS) is a rare, autosomal recessive neurodegenerative pathology, the prevalence of which is estimated at 1/830,000 people (1/55,000–1/700,000 according to Barrett) (Barrett and Bundey, 1997). WFS1 is the main gene involved, responsible for type 1 WS (WS1) (MIM #222300), while the CISD2 gene is responsible for type 2 WS (MIM #604928) (Amr et al., 2007, Inoue et al., 1998). WS1 is characterized by the association of insulin-dependent diabetes and optic atrophy, both occurring before the age of 16 according to EuroWABB guidelines (EuroWAAB, 2014). Other symptoms may occur, such as diabetes insipidus, sensorineural hearing loss, neurological damage, and urinary tract abnormalities (Karzon et al., 2018, Washington University Wolfram Study Group, 2013, Urano, 2016). These vesico-sphincter disorders during WS1 are linked to multiple factors: neurological disorders responsible for a neurological bladder, diabetes insipidus, insulin-dependent diabetes (Ribière et al., 2013, Rove et al., 2018). These well-known disorders are subjects of numerous studies that describe them or discuss their management.

Surprisingly, the French patient association reported to our rare disease center dedicated to Wolfram syndrome that some patients presented with ano-sphincteric dysfunction, which they had never dared to discuss. These symptoms were never mentioned because the patients and their family were not specifically sought during consultations and were very embarrassed. This condition must be considered as debilitating for the patients, who are deeply ashamed of it. Furthermore, these patients believed that these symptoms should not be linked to their condition since they are not part of the "classic list" of symptoms and were never brought up at family meetings. A preliminary study was conducted based on a questionnaire sent to patients with Wolfram syndrome followed in our rare disease center or to patients, who attended to therapeutic education sessions. In this study, 45.95%. of the WS1 patients complained with such severe and disabling problem (Orssaud, 2025). The importance of this frequency allowed us to conclude that these ano-sphincteric disorders are a real and new symptom of WS. It must be taken into account by physicians and treated as such.

To date, there is no clear data to determine the mechanism(s) involved in explaining the occurrence of this sphincter disorder during WS. However, it is essential to differentiate the mechanism and the location of the damage leading to these different causes of ano-sphincteric disorders. In the absence of studies on this subject, we are reduced to making hypotheses concerning these physio pathogenic mechanisms.

## Scientific rationale

In a preliminary study that we conducted, a questionnaire was sent to 37 patients with Wolfram syndrome followed by our department or who had only participated in therapeutic education sessions (Orssaud, 2025). These patients come from all over the country. We received 25 answers. Among those 25 answers, 17 patients reported fecal incontinence and constipation (68.00%). However, to avoid bias, we considered that people who did not respond did not have a fecal incontinence disorder. Thus, the percentage of patients suffering from fecal incontinence and constipation was 45.95% (Orssaud, 2025). The severity of this disorder, evaluated by patients, is high, rated at 8.23 + /- 2.86 on an analogic scale (range 2–10). This allows us to confirm that these ano-sphincteric disorders are a real symptom of WS and must be taken into account and treated as such even though this symptom needs to be confirmed by other teams. The management of these ano-sphincteric disorders is difficult. It requires methods ranging from the simplest dietary advice to more complex techniques for restoring transit control in the literature (Coggrave et al., 2014, Emmanuel, 2010). Understanding this mechanism of this fecal incontinence is not trivial and will have an impact on the therapies that may or may not be offered. This is a reason for justifying the present study.

It is important to determine whether the impairment of anal sphincter control is located in the terminal portion of this pathway or higher up in the brainstem. Knowledge of this location is mandatory to help further specific treatment development.

Neuroradiological studies have clearly demonstrated that brain-stem atrophy is highly possible since patients present with such degenerative involvement (Lugar et al., 2019). According to clinical data, reduction of pons volume, is responsible for swallowing disorders and sleep apnea (Licis et al., 2019). That is why we would look for these symptoms. The hypothesis is that a greater frequency of symptoms should indicate that ano-sphincteric disorders are the consequence of a reduction of pons volume. There will be no difficulty detecting swallowing disorders and sleep apnea. These symptoms are frequently encountered and patients report them spontaneously. They are usually worried about swallowing disorders. In addition, sleep apnea is responsible for chronic fatigue reported by patients, as well as non-restorative sleep. Therefore, the risk of overlooking either of these disorders and any potential association will be limited.

We will not focus on other symptoms such as endocrine disorders with delayed puberty. These symptoms require precise measurements, which are often not performed. Furthermore, these abnormalities tend to point more towards pituitary involvement. Yet, it is not certain that such involvement would be accompanied by damage to the neurological pathways. We will only look for the existence of diabetes insipidus due to associated hydro-electrolytic imbalances. Such imbalances have a potential role in altering stool consistency and may be associated with changes in bowel movements.

The second hypothesis that we will test is that the neurological damage leading to ano-sphincteric disorders could be located in the lower part of the spinal cord (Preziosi et al., 2013). According to literature, such location will lead to an impairment of voluntary control of the anal sphincter due to a reduction of the perception of anorectal fullness which facilitates sphincter control and anticipation of defecation needs (Magnuson et al., 2023, Preziosi et al., 2013). This hypothesis is supported by a similarity of complaint between ano-sphincteric disorders and urinary disorders as reported by patients. The urgency to defecate, leading to ejection of anal content despite efforts to prevent such disastrous situation, is similar to urination due to engorgement and lack of proper bladder emptying. It constitutes a good relationship to test the hypothesis of a localization of the damage at this level. We expect that if this hypothesis will be confirmed, we will find statistically significant association urinary and anosphincteric disorders without any manifestation of pons atrophy. A limitation of this study will be association of swallowing disorders or sleep apnea and lower urinary tract dysfunction. In such case, we will not be able to distinguish between lesions located high in the brainstem and those affecting the spinal cord. Urinary problems can also be due to reduction of pons volume (Rove et al., 2018).

Finally, there is another hypothesis based on the existence of few specific mutations in the WFS1 gene leading to a particular SW1 phenotype with anosphincteric disorders. Lee et al. have clearly shown that the type of mutation can modify the severity of symptoms linked to the WFS1 gene (Lee et al., 2023).

In case of the absence of clear statistically significant association allowing to evoke a degenerative neurological mechanism, we will consider a last hypothesis. There is a rational based on the literature to consider that anosphincteric disorders could be related to dysautonomic disorders observed in childhood type 1 diabetes (Roche et al., 2005, Tang et al., 2013).

## Patients and methods

### Patients

We will conduct a retrospective study based on the analysis of files of patients followed in the Reference Center for Rare Diseases in Ophthalmology since 2018. We will only retain files in which the presence or absence of sphincter disorders is clearly mentioned. Although this symptom is systematically sought during consultations and day hospitalization by the referring physician of the Centre, there is a risk that some patients may not have declared it.

## Method

The method we intend to implement will be based on data from literature concerning the possible neurological origin of fecal incontinence. A neurological mechanism will be considered given the pathophysiology of WS, which is a neurodegenerative condition (Barrett et al., 1993, Barrett and Bundey, 1997, Urano, 2016). It is well known that neurological or ENT symptoms, such as swallowing disorders, will appear during the course of WS (Marshall et al., 2013). However, in order not to overlook variations related to patient mutations, we will add to this study a genetic component. Marshal and other authors demonstrated that the severity of the symptoms during WS is related to the type of mutations harbored by the patients (Marshall et al., 2013)^.^.

In order to determine the mechanism of ano-sphincteric disorders, we will divide the patients into two subgroups according to the presence or absence of ano-sphincteric disorders.

According to this generic mechanism, we will collect different parameters that will be used in the analyses: the duration of the WS, the duration of insulin type I diabetes, the values of glycosylated hemoglobins over this period, the age of patients as well as their visual acuity at first occurrence of ano-sphincteric disorders and the presence of vesico-sphincter disorders, swallowing disorders or sleep apnea (Hoekel et al., 2014).

Firstly, we intend to characterize the severity of the WS in each patient as the occurrence of ano-sphincteric disorders. To that end, we have planned to consider more specifically three parameters from patient’s files for a multi-factorial analysis. We will consider the age of our patients when ano-sphincteric disorders were mentioned for the first time as well as the duration of WS and the duration of insulin type I diabetes at the time of the first mention of this symptom. This duration will be calculated from the age of the first symptom of WS, whatever it may be. These data will be based solely on patient self-reporting. We have no way to increase the reliability of this data. However, this limitation will be taken into account in the analysis. We will collect the LogMAR far best corrected visual acuity at four or one meters according to the number of letters read.

Furthermore, we will conduct statistical tests to investigate a correlation between the presence of swallowing disorders, vesico-sphincter disorders, and sleep apnea and the ano-sphincteric disorders in order to determine if there is a neurodegenerative cause and specify its location as mentioned previously.

In addition, we will study and compare the type of mutations in the WFS1 gene in each patient within the two subgroups since it seems that the severity of WS can vary according to this factor ^7,16^. These mutations are recorded in the patients' files. Therefore, this study will not require any new genetic testing. At least, if the previous analyses are inconclusive, we will compare the duration and severity of diabetes between the groups.

Determining the pathophysiological mechanism of this symptom is essential in order to be able to propose more suitable therapies in the future to improve the quality of life of patients.

## Statistics

Statistical analyses will be performed using SPSS (IBM SPSS Statistics, version 29.0). The primary statistical analysis is based mainly on the study of quantitative data, expressed as means. From this perspective, the normal distribution of quantitative data will be tested with Shapiro-Wilk test. When following a normal distribution, mean comparison will be performed using unpaired Z-test according to the sample size. In case of non-normal distribution, a non-parametric Wilcoxon test will be performed. Generalized linear models were used for the analysis of data including both eyes of the same patient. Binary symptom associations or multiples symptom associations will be tested using Chi-square analysis.

Gven the small sample size, a univariate analysis will be performed as an exploratory evaluation, tacking into account four parameters: existence of vesico-sphincter disorders, swallowing disorders, sleep apnea and diabetes insipidus. In addition, multivariate analysis of variance, multivariate regression and canonical correlation analysis will be performed to evaluate the severity of the WS according to the duration of the WS, the duration of insulin type I diabetes, the age of patients as well as their visual acuity at first mention of ano-sphincteric disorders. We will extend this multi-factorial analysis to look for association between WS severity and neuro degenerative symptoms swallowing disorders, sleep apnea and diabetes insipidus and a Pearson correlation will be achieved between these parameters. We hope that these statistical analyses will allow us to determine whether there is an association between the ano-sphincteric disorders and either of these parameters.

P values less than 0.05 were considered statistically significant and for multiple comparisons a single confidence interval with a 95% will be used for the analysis of the results.

## Ethics statement

According to national rules, patients were previously informed that their data could be anonymized and used for such type of study and their consent was considered as obtained if they did not specify their opposition.

The Ethic Committee of the French Ophthalmological Society approved this retrospective study, which adheres to the tenets of the Declaration of Helsinki. It is registered in Clinical trial under the number NCT07313085.

## Conclusion

WS is a neurodegenerative disease. Therefore, it is logical to assume that the origin of the ano-sphincteric disorders is linked to a reduction in pons volume or to damage to the sphincter control pathways in their terminal portion. However, the type of mutation, which explains the severity of the disease, could also be a factor. Finally, dysautonomic disorders may be considered. In any case, the management of these ano-sphincteric disorders is difficult. in the literature it requires methods ranging from the simplest to more complex techniques for restoring transit control (Emmanuel, 2010). Understanding the mechanism of this symptom is not trivial and will have an impact on the therapies that may or may not be offered.

## CRediT authorship contribution statement

**C. Orssaud:** Conceptualization, Methodology, Investigation, Formal analysis, Validation, Writing – original draft, Writing – review & editing, Project administration.

## Funding

This research did not receive any specific grant from funding agencies in the public, commercial, or not-for-profit sectors.

## Conflict of interest statement

Academic therapeutic research protocols. Travel paid by the French Association of Wolfram Syndrome for symposiums.
